# Rapid Utilization of Telehealth in a Comprehensive Cancer Center as a Response to COVID-19: Cross-Sectional Analysis

**DOI:** 10.2196/19322

**Published:** 2020-07-06

**Authors:** Peter E Lonergan, Samuel L Washington III, Linda Branagan, Nathaniel Gleason, Raj S Pruthi, Peter R Carroll, Anobel Y Odisho

**Affiliations:** 1 Department of Urology Helen Diller Family Comprehensive Cancer Center University of California, San Francisco San Francisco, CA United States; 2 Telehealth Resource Center University of California, San Francisco San Francisco, CA United States; 3 Center for Digital Health Innovation University of California, San Francisco San Francisco, CA United States; 4 Division of General Internal Medicine University of California, San Francisco San Francisco, CA United States

**Keywords:** health informatics, telehealth, video visits, COVID-19, video consultation, pandemic, electronic health record, EHR

## Abstract

**Background:**

The emergence of the coronavirus disease (COVID-19) pandemic in March 2020 created unprecedented challenges in the provision of scheduled ambulatory cancer care. As a result, there has been a renewed focus on video-based telehealth consultations as a means to continue ambulatory care.

**Objective:**

The aim of this study is to analyze the change in video visit volume at the University of California, San Francisco (UCSF) Comprehensive Cancer Center in response to COVID-19 and compare patient demographics and appointment data from January 1, 2020, and in the 11 weeks after the transition to video visits.

**Methods:**

Patient demographics and appointment data (dates, visit types, and departments) were extracted from the electronic health record reporting database. Video visits were performed using a HIPAA (Health Insurance Portability and Accountability Act)-compliant video conferencing platform with a pre-existing workflow.

**Results:**

In 17 departments and divisions at the UCSF Cancer Center, 2284 video visits were performed in the 11 weeks before COVID-19 changes were implemented (mean 208, SD 75 per week) and 12,946 video visits were performed in the 11-week post–COVID-19 period (mean 1177, SD 120 per week). The proportion of video visits increased from 7%-18% to 54%-72%, between the pre– and post–COVID-19 periods without any disparity based on race/ethnicity, primary language, or payor.

**Conclusions:**

In a remarkably brief period of time, we rapidly scaled the utilization of telehealth in response to COVID-19 and maintained access to complex oncologic care at a time of social distancing.

## Introduction

The emergence of the coronavirus disease (COVID-19) pandemic in the United States in March 2020 created unprecedented challenges in the provision of scheduled health care and, in particular, ambulatory cancer care. The rapid spread of COVID-19 has renewed focus on telehealth [1], including video consultations [2], as a means of continuing ambulatory care without increasing the risk of potential exposure for patients, clinicians, and staff.

Telehealth is the provision of health care remotely by means of a variety of telecommunication platforms such as messaging, audio, and video [3]. While the use of telehealth to deliver cancer care is not new [4] and has already been well described [5], delivering it at the current scale as a result of the COVID-19 pandemic is unprecedented.

University of California, San Francisco (UCSF) Health established a telehealth program in 2015, which offers video visits in all practices. In response to the evolving pandemic, leadership challenged the organization to transition all in-person clinic visits, beginning March 15, 2020, to video visits with exceptions only for specific, urgent cases.

In this study, we analyze the change in video visit volume at the UCSF Comprehensive Cancer Center in response to COVID-19 and compare demographics and appointment data from January 1, 2020, and in the 11 weeks after the transition to video visits.

## Methods

**Figure 1:**
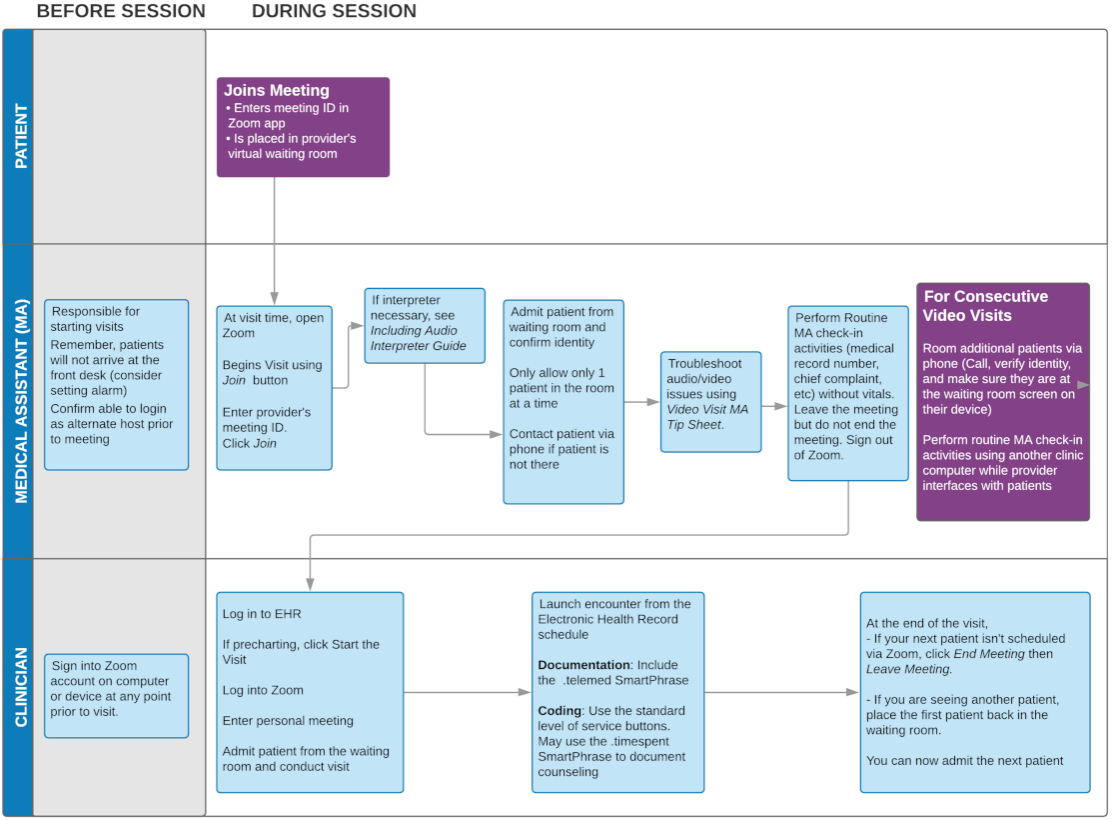
Video visit workflow used at the University of California, San Francisco.

Demographics and appointment data (dates, visit types, and departments) were extracted from the electronic health record reporting database. The pre–COVID-19 period was defined as the 11 weeks from January 1 to March 13, 2020, prior to the transition, and the post–COVID-19 period as the 11 weeks following the transition to video visits on March 16, 2020, up to May 31, 2020. All video visits were performed using a HIPAA (Health Insurance Portability and Accountability Act)-compliant video conferencing platform (Zoom Video Communications Inc) with a pre-existing workflow (Figure 1). The statistical program R (version 3.5.3, R Foundation for Statistical Computing) was used for analysis [6] and a *P* value less than .05 was considered significant.

## Results

**Figure 2:**
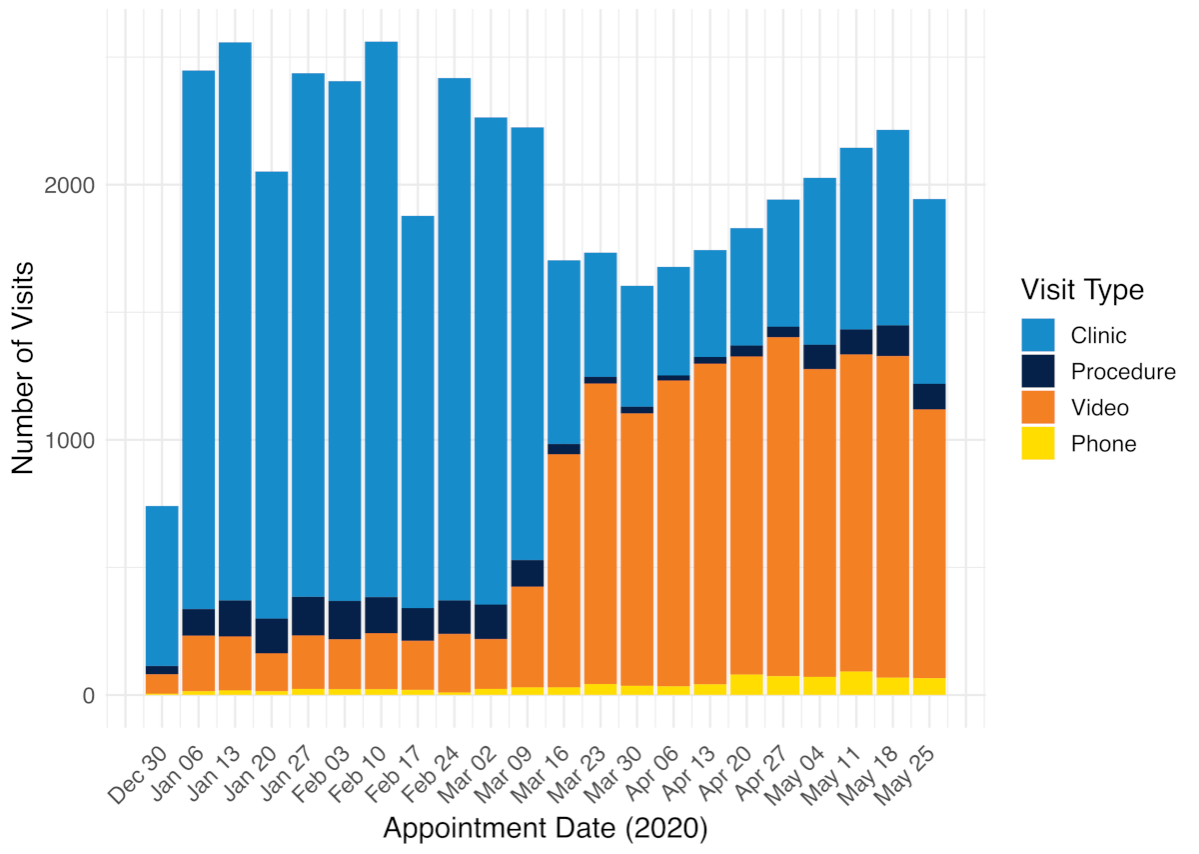
Number of clinic encounters stratified by visit type (in-person visits, procedural visits, video visits, and phone visits) from January 1 to May 31, 2020, with March 16, 2020, denoting the institution-wide transition to video visits in response to coronavirus disease (COVID-19).

**Figure 3:**
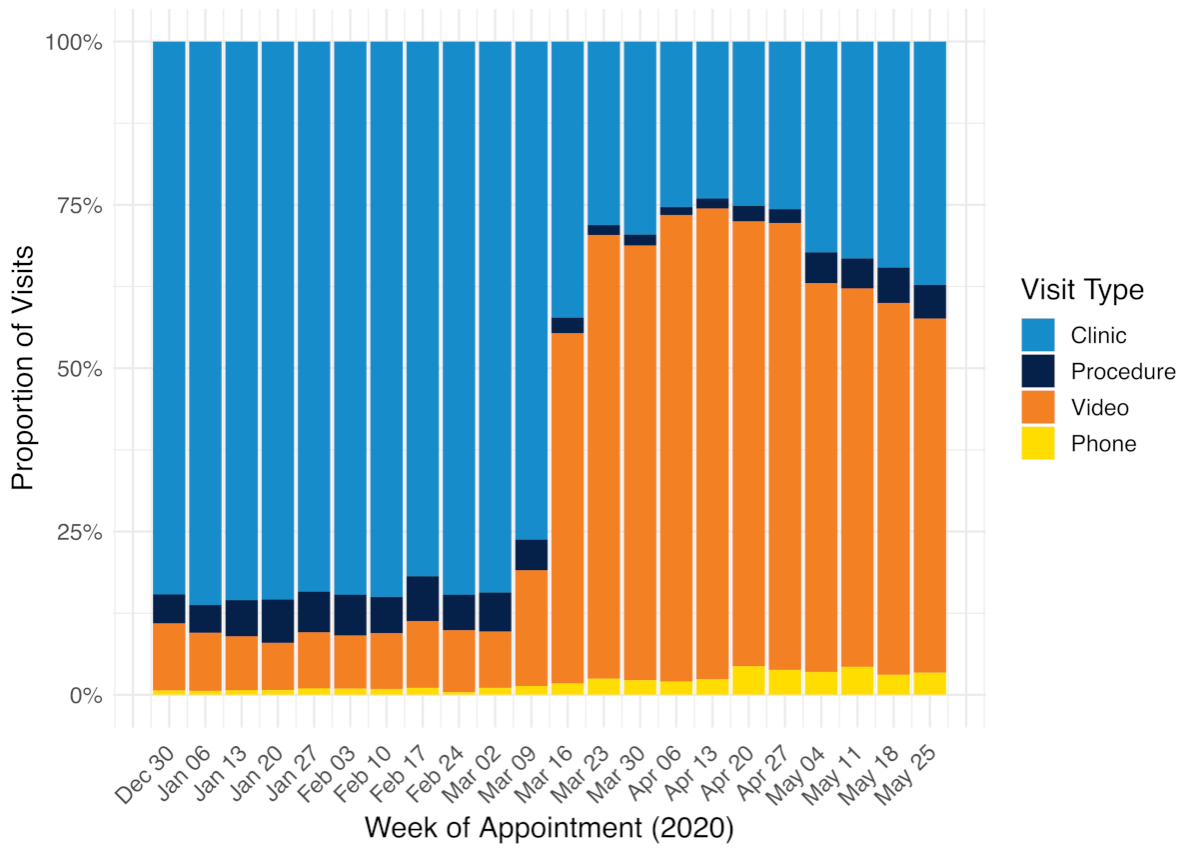
Relative weekly trends in clinic encounters, stratified by visit type (in-person visits, procedural visits, video visits, and phone visits) from January 1 to May 31, 2020, with March 16, 2020, denoting the institution-wide transition to video visits in response to coronavirus disease (COVID-19).

In the pre–COVID-19 period from January 1 to March 13, 2020, there were a total of 23,988 ambulatory care episodes, with a mean of 2181 (SD 522) episodes per week across 17 departments and divisions at the UCSF Cancer Center (Figure 2). During this period, 2284 video visits were performed, with a mean of 208 (SD 75) video visits being performed per week. The proportion of video visits ranged from 7%-18% whereas the proportion of in-person visits ranged from 76%-86% (Figure 3). In the post–COVID-19 period from March 16 to May 31, 2020, there was a total of 20,567 ambulatory care episodes (mean 1870, SD 200 per week). A total of 12,946 video visits were performed in the post–COVID-19 period (mean 1177, SD 120 per week). The proportion of video visits increased to 54%-72%. The proportion of episodes during which a procedure was performed ranged from 4%-7% in the pre–COVID-19 period and 1%-5% in the post–COVID-19 period.

Table 1 displays the demographic data of patients who had a video visit in the pre–COVID-19 period (n=2284) and patients who had a video visit in the post–COVID-19 period (n=12,946). In the post–COVID-19 period, more black/African American (531 [4.1%] vs 78 [3.4%]; *P*<.001), Hispanic/Latino (1450 [11.2%] vs 215 [9.4%]; *P*<.001), and Asian (1903 [14.7%] vs 197 [8.6%]; *P*<.001) patients received care via video visits compared to the pre–COVID-19 period. There was increased post–COVID-19 utilization of video visits for patients in urban areas (12,014 [92.8%] vs 2026 [88.7%]; *P*<.001). We did not find any difference in the insurance status of patients using video visits during either period. In the post–COVID-19 period, first clinic encounter (2822 [21.8%] vs 417 [18.3%]; *P*<.001) and physician-provided visits (10,590 [81.8%] vs 1538 [67.3%]; *P*<.001) increased.

**Table 1:** Demographics of patients who had video visits pre– and post–COVID-19.

Characteristic		Pre–COVID-19 period (n=2284)	Post–COVID-19 period (n=12,946)	*P* value
Age (years), median (IQR)		64.3 (54.9-71.5)	63.6 (52.8-71.8)	.11
Female, n (%)		844 (37)	6123 (47.3)	<.001
**Ethnicity/race, n (%)**				<.001
	White	1606 (70.3)	7988 (61.7)	
	Black/African American	78 (3.4)	531 (4.1)	
	Hispanic/Latino	215 (9.4)	1450 (11.2)	
	Asian	197 (8.6)	1903 (14.7)	
	Other/unknown	188 (8.2)	1075 (8.3)	
Primary language: English		2212 (96.9)	11,962 (92.4)	<.001
Interpreter requested		84 (3.7)	945 (7.3)	<.001
**Marital status, n (%)**				.34
	Married/partnered	1580 (69.2)	8415 (65.0)	
	Single/separated	373 (16.3)	2369 (18.3)	
	Unknown/declined	108 (4.7)	583 (4.5)	
**Patient residence, n (%)**				<.001
	Urban	2026 (88.7)	12,014 (92.8)	
	Rural	255 (11.2)	919 (7.1)	
**Payor, n (%)**				.14
	Commercial	929 (40.7)	5398 (41.7)	
	Medicare	1077 (47.2)	5709 (44.1)	
	Medicaid	224 (9.8)	1541 (11.9)	
	Self-pay	32 (1.4)	155 (1.2)	
First clinic encounter		417 (18.3)	2822 (21.8)	<.001
First video visit		1054 (46.2)	5865 (45.3)	.51
**Provider type, n (%)**				<.001
	Physician	1538 (67.3)	10,590 (81.8)	
	Advanced practice provider	746 (32.7)	2356 (18.2)	

## Discussion

### Principal Findings

We demonstrate a rapid expansion (from <20% to 72%) in telehealth use in a comprehensive cancer center over a remarkably brief time period in response to COVID-19 without differences in race or insurance type. Medicare telehealth visits have increased by more than 25% annually for the past decade [7], yet absolute adoption numbers remain low and fragmented with concerns about potentiating disparities in health care access [8]. The vast majority of cancer care cannot be delayed and the COVID-19 pandemic has presented new challenges that telehealth is uniquely situated to solve. Changes that would typically encompass months of planning, pilot testing, and education have been compressed into days. The use of telehealth has grown exponentially with some practices transitioning to near-complete virtual care in as little as a few days [9-11].

Several factors have likely enabled the rapid expansion of video visits at our institution. First, we had an established telehealth structure and workflow familiar to providers and practice staff. Second, UCSF made a strategic decision to provide work Relative Value Unit (wRVU) credit to providers for telehealth visits since 2015, irrespective of payer reimbursement. Third, new regulatory changes at the federal and state levels as a response to COVID-19 have reduced barriers, including the ability to see new patients (including Medicare beneficiaries) without a prior in-person visit to establish care and reimbursement for telehealth encounters by the Centers for Medicare & Medicaid Services (CMS) at parity with in-person visits beginning March 17, 2020 [12]. Finally, CMS now permits providers licensed in any state to provide telehealth services across the country [13].

### Limitations

There are a number of limitations to this study that need to be acknowledged. We did not evaluate the outcome of the video visits (eg, patient satisfaction and qualitative or clinical outcomes). The study is from a single, large, urban academic cancer center in the United States and our findings may not be generalizable to other specialties, practices, or locations. We believe that this is the first report of the utilization of video visits in a comprehensive cancer center in response to COVID-19 with a detailed description of the changing demographic of patients utilizing video visits before and after the COVID-19 pandemic.

### Conclusions

Overall, the proportion of video visits increased from 7%-18% to 54%-72% between the pre– and post–COVID-19 periods while maintaining access to complex oncologic care at a time of social distancing. The COVID-19 pandemic has forced us to radically rethink and change our cancer care delivery models. In many health systems, there will undoubtedly be many lessons learned from this “natural experiment,” which has the potential to permanently change care delivery patterns.

## Abbreviations

CMS: Centers for Medicare & Medicaid Services
COVID-19: coronavirus disease
HIPAA: Health Insurance Portability and Accountability Act
UCSF: University of California, San Francisco
wRVU: work Relative Value Unit
